# Evaluation of Serum Testosterone Levels Following Three Months of SA3X (Spilanthes acmella) Supplementation

**DOI:** 10.7759/cureus.26236

**Published:** 2022-06-23

**Authors:** Nabnita Patnaik, Kumar Guru Mishra, Nihar Ranjan Pradhan

**Affiliations:** 1 Obstetrics and Gynecology, All India Institute of Medical Sciences, Bibinagar, Hyderabad, IND; 2 Community Medicine and Family Medicine, All India Institute of Medical Sciences, Bibinagar, Hyderabad, IND; 3 Vascular and Endovascular Surgery, Apollo Hospitals, Hyderabad, IND

**Keywords:** supplement, testosterone (tt), men’s health, erectile dysfunction, spilanthes acmella

## Abstract

Introduction: Low testosterone is usually associated with erectile dysfunction (ED). SA3X (*Spilanthes acmella*) has proven to be effective in alleviating symptoms of ED, which could be due to an alteration in serum testosterone levels. This study was carried out to evaluate the change in testosterone levels in participants with ED supplemented with SA3X for three months.

Materials and Methods: A group of 326 sexually active men aged 25-60 years was investigated from November 2021 to May 2022 in Hyderabad. The participants were subjected to supplementation with SA3X capsules for three months, and a follow-up was done at the end of six months with serum testosterone assessment in each visit. The change in testosterone level was assessed using a mixed model repeated measures analysis.

Results: A significant increase was observed in the mean serum testosterone levels by the end of the second month (323.91 ± 13.76 ng/dL vs. 309.84 ± 14.11 ng/dL; p=0.03) and third month (332.27 ± 12.85 ng/dL vs. 309.84 ± 14.11 ng/dL; p<0.01) of SA3X therapy. The adjusted mean change in testosterone levels was found to be 22.43 ng/dL at the end of the three-month therapy. It was also observed that the change in testosterone levels was significantly lower in participants having diabetes mellitus, hypercholesterolemia, and a history of substance abuse. However, participants on phosphodiesterase-5 inhibitors had an increased change in testosterone levels.

Conclusion: Supplementation with SA3X capsules for three months increases the serum testosterone levels. However, causality cannot be ascertained owing to the longitudinal nature of the study, and further controlled trials are required for the same.

## Introduction

Testosterone is the primary androgenic hormone seen in both the male and female sex. Normal testosterone levels in males are required to maintain secondary sexual characteristics, fertility, muscle mass, hair growth, and sexual function [1-2]. As part of natural male aging, there is generally a decrease in testosterone levels secondary to diminished gonadal function [3]. In conjunction with testosterone reduction in men, a decline in erectile function is often seen [4-5]. Low testosterone is usually associated with erectile and sexual dysfunction in illnesses like metabolic syndrome, demonstrating the relevance of testosterone in sustaining erectile function [5-6].

As testosterone levels steadily decrease in diabetes, hypercholesterolemia, and central obesity, patients will encounter and exhibit failure to achieve adequate penile tumescence and often experience a concurrent reduction in sexual desire [7-8]. The same remains applicable for those on oral hypoglycemic agents, anti-hypertensives, statins, anti-depressants, and other drugs [9-11]. Outside of such disorders as metabolic syndrome, healthy elderly males can also experience similar abnormalities [12]. During normal aging, the increased prevalence of sexual and erectile dysfunction (ED) with hypogonadism further illustrates a possible correlation [3, 13].

*Spilanthes acmella* has proven to be effective in alleviating symptoms of ED; however, the mechanism of action is yet to be determined in humans [14-15]. SA3X capsules, which contain 500 mg of *S. acmella* extract standardized to 3.5% spilanthol, yielding 17.5 mg of spilanthol, have been introduced by Stiriti Ayur Therapies Pvt. Ltd., India.

Given the relationship between testosterone and erectile function, it can be determined that testosterone levels impact the functioning of male sexual health. Thus it is vital to evaluate the effects of SA3X on the serum testosterone levels. This longitudinal study was thus carried out to evaluate the change in testosterone levels in participants with ED supplemented with SA3X capsules for three months.

## Materials and methods

A group of 326 sexually active men ranging in age from 25 to 60 years was investigated from November 2021 to May 2022. The study included community-dwelling men with complaints of ED for one month or more attending the outpatient departments of Apollo Hospitals, Hyderabad, irrespective of their comorbid status and history of substance abuse. Cases with serious medical illnesses -- including but not limited to cancer, HIV-AIDS, and those on immunosuppressive agents, requiring hospitalization -- were excluded from the study. Ethical approval for the study was obtained from Institutional Ethics Committee -- Biomedical Research, Apollo Hospitals, Hyderabad (AHJ-ACD-045/03-21), and the study was conducted per the Declaration of Helsinki. All study participants were fully informed about the aim of this study and gave written consent to participate in the investigation.

Baseline assessment

The participants underwent an anthropometric evaluation and blood examination to determine their serum testosterone levels. Participants responded to the International Index of Erectile Function (IIEF) questionnaire [16]. The participants were also asked to note the time (in minutes) they could maintain a penile erection without being flaccid in the first instance of sexual arousal for the last seven days, and the average duration was determined.

Study medication/supplementation

The participants were subjected to supplementation with SA3X capsules for three months, and a follow-up was done at the end of six months. The serum testosterone level was assessed during each visit -- the end of the first, second, third, and sixth months. Overnight blood samples from the antecubital vein were obtained at rest between 7 AM and 11 AM for all participants. Serum testosterone measurements were performed using liquid chromatography-tandem mass spectrometry (LC-MS/MS) with detection limits of 6.63 ng/dL in a central laboratory accredited by the Indian Council of Medical Research. The intra- and interassay coefficients of variation were <11%. The participants were grouped into two categories based on the serum testosterone levels -- Group A having serum testosterone < 300 ng/dL and Group B having serum testosterone ≥ 300ng/dL -- to observe whether the baseline serum testosterone levels influence the change in levels following administration of SA3X capsules.

Statistical analysis

The sample size was determined by the expected change in total serum testosterone levels, which was the study's primary outcome measure. The study was powered to detect a five‐unit treatment difference. Assuming a common SD of 15, power=80%, alpha level=0.05, and loss to follow-up of 20%, 290 patients were needed. A mixed model repeated measures (MMRM) analysis was used to analyze the changes in serum testosterone levels from baseline. We created linear regression models to determine the independent relationship between change in serum testosterone levels and baseline parameters. Only variables with an association having p < 0.2 entered the multiple linear regression model. The significance level was fixed at p ≤ 0.05, and all data were presented as the mean ± SD or percentages. The analyses were performed using IBM SPSS Statistics for Windows, version 26 (IBM Corp., Armonk, NY, USA).

## Results

Basic characteristics

The basic anthropometric, IIEF scores, serum testosterone levels, and personal characteristics of the studied group are given in Table 1. The group consisted of both sedentary and active men, and the actual mean level of physical activity amounted to 3.1 ± 1.8 hours per week. As shown in Table 1, the two groups -- A & B, significantly differed (p = 0.02) only in serum testosterone levels.

**Table 1:** Summary of baseline characteristics of the study participants. Group A – Participants with serum testosterone < 300 ng/dL at recruitment Group B – Participants with serum testosterone ≥ 300 ng/dL at recruitment BMI, body mass index; IIEF, International Index of Erectile Function; PDE5i, phosphodiesterase-5 inhibitors; OHA, oral hypoglycemic agents; HTN, hypertensive; SD, standard deviation *p < 0.05

	Total (N = 326)	Group A (N = 181)	Group B (N = 145)	p-value
Age (years), mean ± SD	46.17 ± 8.19	47.66 ± 6.16	43.16 ± 9.12	0.10
BMI (kg/m^2^), mean ± SD	27.77 ± 4.18	28.98 ± 4.22	26.18 ± 5.98	0.18
Duration of penile erection (minutes), mean ± SD	1.65 ± 0.51	1.62 ± 0.88	1.74 ± 0.27	0.28
Serum testosterone (ng/dL), mean ± SD	309.84 ± 14.11	252.67 ± 10.91	381.21 ± 18.11	0.02*
Baseline IIEF score, mean ± SD	18.16 ± 4.64	18.12 ± 5.21	18.22 ± 4.19	0.44
Comorbid conditions, n(%)				
Diabetes mellitus	179 (54.91)	92 (50.82)	87 (60.00)	0.09
Hypertension	158 (48.46)	83 (45.85)	75 (51.72)	0.29
Hypercholesterolemia	91 (27.91)	54 (29.83)	37 (25.52)	0.38
Cardiovascular disease	29 (8.89)	19 (10.49)	10 (6.89)	0.25
Thyroid disorders	8 (2.45)	6 (3.31)	2 (1.37)	0.26
Concomitant medications, n(%)				
PDE5i	95 (29.14)	56 (30.93)	39 (26.89)	0.42
OHAs	166 (50.92)	86 (47.51)	80 (55.17)	0.16
Anti-HTNs	145 (44.48)	75 (41.43)	70 (48.27)	0.21
Statins	82 (25.15)	48 (26.51)	34 (23.44)	0.52
Anti-depressants	33 (10.12)	20 (11.05)	13 (8.96)	0.53
Substance abuse, n(%)				
Alcohol	65 (19.93)	39 (21.54)	26 (17.93)	0.41
Smoking	95 (29.14)	56 (30.93)	39 (26.89)	0.42
Other drugs	8 (2.45)	6 (3.31)	2 (1.37)	0.26

Change in testosterone levels

**Figure 1:**
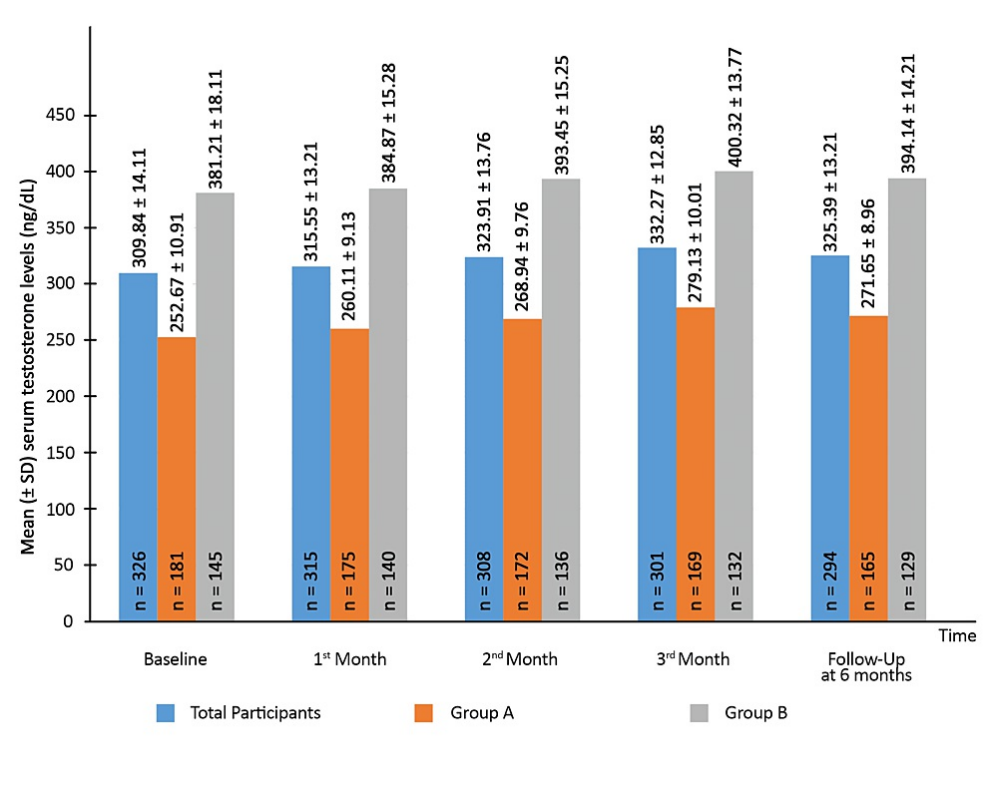
Mean change in serum testosterone levels over the study duration. Group A – Participants with serum testosterone < 300 ng/dL at recruitment Group B – Participants with serum testosterone ≥ 300 ng/dL at recruitment

The SA3X capsules were advised as a supplementary therapy to the subjects for three months attending the OPD at baseline. A significant increase was observed in the mean serum testosterone levels by the end of the second month (323.91 ± 13.76 ng/dL vs. 309.84 ± 14.11 ng/dL; p=0.03), and a further increase was seen by the end of the third month (332.27 ± 12.85 ng/dL; p<0.01). After discontinuing therapy, the testosterone levels were measured again at the end of the sixth month. The difference from baseline was found to be statistically significant (325.39 ± 13.21 ng/dL vs. 309.84 ± 14.11 ng/dL; p= 0.04). The same was the case for Groups A and B, as depicted in Figure 1. 

**Figure 2:**
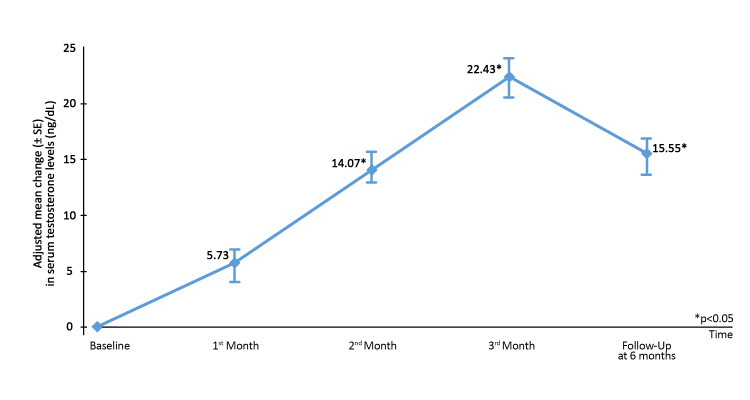
Adjusted mean (±SE) change in serum testosterone levels in study population from baseline till the third month of treatment and follow-up after three months of discontinuation of treatment. *p < 0.05 SE, standard error

Figure 2 shows a significant change in the adjusted mean change in serum testosterone levels between the end of the second month, the third month, and the sixth month vs. baseline (p<0.05).

Multivariate analysis

In order to evaluate parameters that could affect the change in serum testosterone levels, a multivariate linear regression analysis was carried out. Body mass index (BMI) was found to have a significant negative association with the change in testosterone levels. It was also observed that the change in testosterone levels was significantly lower in participants having diabetes mellitus (-14.87 ng/dL; p=0.02) and hypercholesterolemia (-12.12 ng/dL; p=0.02) in comparison to those without diabetes and hypercholesterolemia. However, it was found that the change in testosterone was not influenced by the baseline levels of testosterone (p=0.87). The other parameters having a significant association with the change in testosterone levels have been outlined in Table 2.

**Table 2:** Multivariate regression analysis between baseline parameters and change in testosterone levels over three months. All the explanatory variables (age, BMI, duration of penile erection, serum testosterone levels, IIEF score, comorbid conditions, and concomitant variables) entered the regression model, and only those with p < 0.2 were included in the final model. SEM, standard error of mean; BMI, body mass index; PDE5i, phosphodiesterase 5 inhibitors; OHA, oral hypoglycemic agents *test statistic; †p value < 0.05; ‡regression coefficient

Outcome variables	Parameters	B*	SEM	p-value
Change in testosterone levels (ng/dL)	Age (years)	- 0.17	0.29	0.71
	BMI (kg/m^2^)	- 0.75	0.11	0.03^†^
	Diabetes mellitus	- 14.87	5.21	0.02^†^
	Hypertension	- 6.12	5.33	0.11
	Hypercholesterolemia	- 12.12	4.12	0.02^†^
	PDE5i	16.78	5.29	< 0.01^†^
	OHAs	- 13.87	8.76	0.02^†^
	Statins	- 9.65	4.42.	0.03^†^
	Alcohol	- 18.11	6.21	< 0.01^†^
	Smoking	- 20.26	6.19	< 0.01^†^
	Serum testosterone < 300 ng/dL	3.23	1.65	0.87
	Serum testosterone ≥ 300 ng/dL	0^‡^		

## Discussion

The findings presented in this study demonstrate that a three-month supplementation of SA3X in patients with ED leads to an increase in serum testosterone levels by 22.43 ng/dL by the end of the third month. This is supported by findings that even post-discontinuation, the increased levels of testosterone sustained with a 15.55 ng/dL increase at the end of follow-up in the sixth month. Owing to the inclusion of study participants with comorbidities, concomitant medications, and a history of substance abuse, serum testosterone levels are influenced by other parameters, as evident from the regression analysis outlined in Table 2. However, a notable finding is that the change in testosterone levels is adversely affected due to higher BMI, diabetes mellitus, hypercholesterolemia, intake of oral hypoglycemic agents (OHAs), statins, and history of abuse of alcohol and smoking. The sole baseline parameter promoting the change in testosterone levels is the intake of phosphodiesterase 5 inhibitors (PDE5i) along with SA3X capsules. It must also be acknowledged that these results, based on associations, do not infer causation.

According to several pieces of research, serum testosterone levels in men decrease with age. There is a 1% decline per year, as evident in cross-sectional research, whereas longitudinal studies show a more drastic decrease. [17]. Testosterone concentration has been proven to be lower in men who are obese [18-20] and who have aging-associated comorbidities such as diabetes [21]. The current study reverberates similar findings with persons having comorbidities showing a decreased change in testosterone levels following SA3X therapy.

In a review by Sansone et al. [22], it has been mentioned that smoking, alcohol use, and recreational drug consumption impair male fertility, with possible synergistic, rather than additive, effects. In humans and animal models, spermatogenesis and sperm parameters have been impaired along with increased DNA methylation and oxidative stress; similarly, effects on endocrine control of reproductive and sexual function have been described in clinical and experimental research. A similar association of decreased change in testosterone levels was observed in this study. This study also corroborates the findings of a review by Spitzer et al. [23] where it was shown that administration of an optimized dose of PDE5i to men with ED and low baseline serum testosterone increases serum testosterone levels likely by a direct action on the testes.

The risks and benefits of testosterone therapy have been reviewed comprehensively in clinical guidelines [24-26]. From the public health perspective, nonpharmacologic strategies to normalize testosterone levels in older men with comorbidities may be preferable to testosterone supplementation because such strategies may benefit overall health and not be associated with adverse effects [27]. Such randomized intervention trials have not been conducted on a large scale, so it remains unclear whether the published data would translate into increases in testosterone if men resorted to those interventions [28]. Spilanthes have been proven to increase testosterone levels in a murine study by Sharma et al. [29]; however, trials on humans claiming the same have not been published yet. Furthermore, this study being of a longitudinal design cannot prove causality [30].

Unfortunately, it was impossible to ensure a controlled environment for the participants in a study of this nature. Whereas our study population is appropriate, the small size of some subgroups reduces the statistical power, and the analysis of these groups becomes less accurate. The blood sample collection for serum testosterone analysis should optimally be done during the morning hours. The same was adhered to in the current study, so this is unlikely to have affected the outcomes.

Our study cannot approximate the causes of the change in serum testosterone levels to SA3X. However, the fact that the changes occurred significantly over a relatively short period despite comorbid conditions and other risk factors -- known to decrease testosterone levels -- suggests that SA3X has an important role. These changes need to be identified further in randomized controlled trials.

## Conclusions

Supplementation with SA3X capsules for three months affects the change in serum testosterone levels positively. However, due to confounding factors such as comorbid conditions, concomitant medications, and history of substance abuse, the increase cannot be ascertained to SA3X only. In order to establish causation, randomized controlled trials must be carried out in a representative sample over various geographical units. 
